# A Critical Look at Critical Periods

**DOI:** 10.1146/annurev-vision-101322-110319

**Published:** 2025-09

**Authors:** Ryan Gorzek, Joshua T. Trachtenberg

**Affiliations:** Department of Neurobiology, David Geffen School of Medicine at UCLA, Los Angeles, California, USA

**Keywords:** cortex, plasticity, vision, critical period, feature detector, complex, ipsilateral

## Abstract

Over the past decade and a half, a new understanding has emerged of the role of vision during the critical period in the primary visual cortex. Rather than driving competition for cortical space, vision is now understood to inform the establishment of feature conjunctions that cannot be constructed intrinsically. Longitudinal imaging studies reveal that the establishment of these higher-order feature detectors is a remarkably dynamic process involving the gain and elimination of neurons from functional groups (e.g., binocular neurons with nonlinear response tuning). Experience exerts its influence selectively on this developing circuitry; some pathways require experience for normal development, while others appear to be intrinsically established. This difference drives the network dynamism that is exploited to construct novel cortical representations that best encode our local environment and inform our actions in it.

## INTRODUCTION

The infant neocortex has the unique capacity to process diverse sensory inputs, enabling infants to differentiate a wide range of stimuli, including phonemes, visual patterns, and tactile inputs, regardless of their relevance to the native environment. Experience refines these circuits, building novel feature conjunctions that are reinforced by the environment while pruning those that are not. This process unfolds during critical periods and sensitive periods, which represent distinct temporal windows of heightened learning differentiated by the degree and persistence of environmental influences on neural circuits (Knudsen 2004). During critical periods, experience drives permanent changes to cortical circuitry. These changes are well-characterized in primary sensory cortices, with ocular dominance plasticity in primary visual cortex (V1) being a particularly well-studied example. Sensitive periods are more protracted intervals of heightened plasticity during which environmental inputs continue to significantly shape neural circuits. Changes during sensitive periods are not immutable. The interaction between critical periods and sensitive periods is evident in language acquisition, where languages can be learned well into adulthood, illustrating the graded nature of sensitive periods, but in the absence of exposure during critical periods there is an immutable loss in the ability to distinguish certain phonemes.

How does experience impact cortical structure and function during critical periods? Studies in V1 have defined three primary features of critical periods: There is (*a*) a precritical period during which experience has little impact on cortical circuitry; (*b*) an onset, where something about the cortical circuit changes, and this circuitry is now modified by our experiences; and (*c*) closure, where something about the system changes again, plasticity is lost at key sites, and earlier changes to the circuit are now immutable. Here, we examine the origin of these views of the critical period and suggest alternative interpretations of older data as we place them in the context of newer data. In broad strokes, it is clear that the cortex begins learning about the statistics of the outside world from the moment sensations are conveyed to it (e.g., at eye opening in the case of V1). The activity patterns of these inputs inform the progressive construction of more sophisticated circuits in the upper cortical layers, and these changes serve to maximize feature detection in the local environment. The maturation of inhibition consolidates these changes, as does the loss of plasticity at thalamocortical synapses. In this model, the only defining feature of the critical period is its closure and resulting permanence. Prior to that, visual cortical plasticity is a continuous process beginning prior to eye opening as spontaneous retinal activity is conveyed to the cortex and continuing uninterrupted after eye opening as vision drives new patterns of retinal activity. It is necessary to note, at the outset, that all aspects of visual cortical development appear to be activity dependent. The focus of this review is not on activity, per se, but on vision and its role in cortical circuit development.

## BACKGROUND

The neocortex excels in abstracting experiences to create generalized rules used for inference and prediction (Hawkins et al. 2009). While cortical circuitry is exceedingly plastic, its learning occurs in a highly constrained neural architecture that is, to a large degree, innately established and hardwired. In utero, the major axes of the cortex—anteroposterior, mediolateral, and dorsoventral—are specified by gradients of morphogens and transcription factors, whose intersection establishes proto-areas (O’Leary 1989, Cadwell et al. 2019). Subsequent thalamic innervation of the cortex gives rise to modality-specific areas, such as primary sensory areas (Vue et al. 2013, Antón-Bolaños et al. 2019), and this innervation is intrinsically guided by graded expression of cell-surface molecules (e.g., ephrins and their Eph receptors in the visual system) (Cang et al. 2005a) and shaped by spontaneous patterns of activity originating in the sensory epithelium (e.g., retinal waves) (Ackman et al. 2012, Ge et al. 2021, Matsumoto et al. 2024). It is through these innate mechanisms that modality-specific maps, such as retinotopy in V1, are established. Even the spatial organization of different types of sensory neurons in the epithelium constrains the kinds of feature detectors that can emerge in the cortex—for example, neurons that respond to particular orientations (Paik & Ringach 2011, Song et al. 2021). Thus, once mammals begin to hear, see, and touch the world, cortical processing of these interactions must work within these constraints to construct a set of feature detectors that optimally extract information about the specific environment.

Primary sensory cortices are not unique in their ability to rapidly learn from experience—all cortical regions are capable of this, and indeed all neural systems are learning systems. What distinguishes primary sensory cortices is their ability to stop learning and to establish immutable memories. Since the transformation of sensory information that first occurs in primary cortices establishes the foundation for higher-order processing in association cortices, the permanence of the circuitry supporting these transformations ensures consistent and accurate perception throughout the mammal's lifespan. By restricting this permanence to only some circuit elements within primary sensory cortices, more limited but advantageous plasticity can be maintained in the remainder of the circuit (Yaeger et al. 2024). There is a downside to this strategy. Should sensory information be degraded during this period, as may occur with damage or disuse of the sensory epithelium, cortical circuitry is also indelibly degraded (Wiesel & Hubel 1963). Indeed, this loss of function and the period of sensitivity to this loss are what define the critical period.

Most of what we know about critical periods is based on studies of ocular dominance plasticity in V1, where disuse of one eye leads to the loss of its cortical representation and the subsequent expansion of the cortical representation of the other eye. However, because ocular dominance and its plasticity have no discernable function (Horton & Adams 2005, Adams & Horton 2006, Scholl et al. 2017, Stephany et al. 2018, Fu et al. 2023), efforts to define ocular dominance plasticity have, in many ways, muddled our understanding of critical periods and the plasticity mechanisms driving them. Here, we examine the predominant model of the critical period in V1, which has been summarized in several reviews (Espinosa & Stryker 2012, Levelt & Hübener 2012, Hensch & Quinlan 2018, Hooks & Chen 2020, Reh et al. 2020), and offer an alternative, grounded in the view that experience profoundly influences the development of some functional pathways within the cortex while minimally impacting others.

## WHAT DOES CORTEX DO?

To understand the role of experience in shaping cortical structure and function, and thus the necessity for critical periods, we need to place the question in an ethologically relevant framework. The six-layered neocortex evolved from a three-layered dorsal pallium (Reiner 1993, Shepherd & Rowe 2017, Tosches & Laurent 2019) around 225 million years ago during the Late Triassic (Novacek 1997, Araújo et al. 2022) as mammals evolved from therapsids following the Permian-Triassic extinction event. This initiated a shift in sensory and motor processing. Using the visual system as an example, early vertebrates such as fish, amphibians, and reptiles process a significant amount of visual information—informing orientation and escape—directly in the retina and the tectum. This processing remains low-level given the proximity of these structures to the sensory epithelium. As the neocortex emerged, it supported learning higher-order representations defined by combinations of multiple visual features, also referred to as feature conjunctions (Murray & Wise 2012). These feature conjunctions supported the emergence of visual affordances (Cisek 2007), which are the potential actions an animal can take based on what they see. In other words, the features of an object determine its uses, and the features of the local scene determine possible actions. These new representations are learned, and they advance the same goal as the earlier nervous system—to keep you alive.

## HOW DO CRITICAL PERIODS SUPPORT THIS?

Staying alive is straightforward in utero, when someone else is keeping you fed and safe. Once you are born, you must rapidly learn how to find food and avoid becoming food in the process. These two pressures—foraging and avoiding predation—drove the evolution of the nervous system, including the mammalian neocortex (Cisek 2022, Osvath et al. 2024). From this point of view, the purpose of the brain is just to execute behaviors that counteract deviations from a desired physiological state, largely set by the hypothalamus (Cisek 2022).

When mammals first emerged, so did several new biological innovations. Foremost among them was endothermy, which included the development of hair or fur and sweat glands (later modified into mammary glands for milk production). Endothermy provided many advantages to early mammals: They could forage at night when temperatures dropped, avoiding predation from cold-blooded predators; they expanded their foraging ranges; and they maintained a more efficient metabolism and greater reproductive success with stable internal temperatures. However, endothermy came with a rather substantial metabolic price tag. At rest, a mammal burns almost ten times as many calories as a similarly sized reptile (Bennett & Ruben 1979), and about twenty times as many in motion. The evolution of the neocortex, with its increased capabilities for integrative processing, prediction, biased competition, and action, supported this high-calorie lifestyle by significantly improving foraging success and efficiency. The addition and expansion of neocortical areas also enhanced sensorimotor processing, partly in the form of new, higher-order conjunctions and affordances. These conjunctions and affordances are informed by our experiences throughout our lifetimes but are built from combinations of feature detectors established and consolidated in primary sensory cortices by our experiences during critical periods.

As discussed above, during critical periods, the kind and quality of our earliest experiences indelibly alter neocortical circuitry in these primary sensory cortices to most accurately process the information found in the local environment. By doing so, critical periods promote foraging success and predator avoidance to a degree that cannot be achieved by genetically hardwired systems.

## CRITICAL PERIODS IN V1

### How Critical Period Plasticity Is Measured: Receptive Field Tuning Properties

Critical periods have been observed in every primary sensory cortex. Most of our understanding of them comes from studies of V1, where they were first identified. This is largely because the receptive field tuning properties of neurons in V1 are well-characterized, and therefore, deviations from normal are readily detected and quantified. Each neuron in V1 responds to stimuli in a particular part of visual space, and this region delineates its receptive field. Receptive field tuning properties refer to how vigorously this neuron responds to distinct features of a visual stimulus, such as its orientation, direction of motion, spatial and temporal frequency, position in the receptive field (response linearity), contrast, size, distance (depth), and whether stimuli outside of this receptive field influence its firing. No single study has characterized how early visual experience impacts all these properties, making it difficult to reach a full understanding of the role of vision in setting up and maintaining these responses. However, we can achieve a more comprehensive understanding by comparing measures made across a range of species where some measures have been exceedingly well-executed in one or the other. The species most often used to study visual cortical development are mice, rats, ferrets, cats, and macaques. All the aforementioned receptive field tuning properties have been found in V1 of these animals, which span two superorders (Laurasiatheria and Euarchontoglires), indicating that they were present in the last common ancestor and are, therefore, homologous features (Van Hooser 2007). Given this, when we examine the critical period below, we draw on studies across these species.

### The Fallacy of an Opening of the Critical Period

The current model of the critical period holds that visual experience has little influence on visual cortical circuitry in rodents and carnivores until about a week after eye opening. That is, there is an opening of the critical period. Or, from a different perspective, the kind and quality of visual experience during this first week after eye opening are not terribly important for shaping cortical circuit properties.

Before proposing an alternative explanation of the observations at the foundation of these conclusions, it is necessary to explore the origins of the current model and where, in our opinion, it would benefit from revision. Critical periods in the cortex were first defined by David Hubel and Torsten Wiesel (Hubel & Wiesel 1963) in their studies of kittens deprived of vision through one eye at varying ages of onset and for varying durations. They, and others (Stryker & Sherk 1975, Sherk & Stryker 1976, Stryker et al. 1978), concluded that most of the structure and function of V1 were intrinsically established and adult-like at birth. They asserted that the role of vision is not to instruct visual cortical circuitry but to maintain it until the critical period closed. In this view, vision has little impact on cortical structure and function until about a week after eye opening in kittens, when it rather suddenly becomes necessary for maintenance (Wiesel & Hubel 1963, Hubel & Wiesel 1970). Similar claims have been made in mice and ferrets. This notion of a precritical period, where vision has little impact on cortical architecture, is deeply entrenched in textbooks and review articles (Hensch 2004, Espinosa & Stryker 2012, Levelt & Hübener 2012, Hooks & Chen 2020) and has now led to a half-century of effort toward identifying the molecular and cellular mechanisms that open critical periods.

On the face of it, the existence of a precritical period seems remarkably unlikely. In all species studied, neurons in V1 respond to visual stimuli shortly after eye opening, often with remarkable precision (Hubel & Wiesel 1963, Blakemore & Van Sluyters 1975, Chapman & Stryker 1993, White et al. 2001, Prévost et al. 2010, Hoy & Niell 2015). Yet, this activity purportedly lacks the ability to inform cortical circuitry. This notion becomes even more perplexing when we consider the fact that prior to eye opening, spontaneous patterns of retinal activity are already capable of informing the structure and function of cortical circuitry (Cang et al. 2005b, Ackman et al. 2012). Thus, to hold onto the view of a precritical period, and with it, the belief that the critical period opens, we must believe that (*a*) retina-driven cortical activity is informative before eye opening (e.g., in the establishment of retinotopy) and again some weeks thereafter, and (*b*) these periods of plasticity are interrupted by a plasticity dead zone that suddenly begins at eye opening.

The field reached these conclusions by comparing ocular dominance scores—a measure of each eye's relative strength in driving cortical neuron responses—in normal animals versus age-matched animals briefly deprived of vision. This approach is problematic for reasons uncovered decades later. Chief among them is that ocular dominance lacks physiological or behavioral significance, and thus measures of ocular dominance plasticity cannot inform our understanding of the behaviorally relevant changes to cortex that occur from the moment the eyes open. We illustrate this by examining the link between ocular dominance and the behaviorally relevant measure of stereopsis. In mammals with forward-facing eyes, both eyes view the same space but capture slightly different images due to horizontal offset. These images converge on binocular neurons in V1, which are tuned to the absolute disparity of the two images and use this difference to extract depth information (Cumming & Parker 1999). Although one might expect the physiological measure of ocular dominance in a given cell to predict the degree of disparity tuning in that cell, it does not (Ohzawa & Freeman 1986, LeVay & Voigt 1988, Freeman & Ohzawa 1990, Read & Cumming 2004, Scholl et al. 2013, Fu et al. 2023). In other words, a cell that is seemingly monocular or overwhelmingly dominated by one eye can have normal binocular disparity tuning. Nor is there any relationship between anatomical measures of ocular dominance—the presence, size, and spacing of ocular dominance columns—and depth perception (Livingstone et al. 1995, Horton & Hocking 1996, Adams & Horton 2006). Even after unilateral vision blur or strabismus, the amblyopic deficits are not correlated with shifts in ocular dominance (Kiorpes 2015). Thus, neither physiological nor anatomical measures of ocular dominance have any obvious behavioral or functional correlate (Livingstone et al. 1995, Horton & Adams 2005, Adams & Horton 2009).

Emergent from the early studies on ocular dominance columns and their plasticity was the view that most aspects of visual cortical structure and function are intrinsically established and that a major role of vision is to drive a competition between the two eyes for cortical representation. Within this view, patterned vision prior to the onset of the critical period does not inform receptive field tuning for either eye, nor the formation of ocular dominance columns, but is required thereafter for the strengthening and maintenance of this selectivity as well as the competitive plasticity (Crair et al. 1998, Espinosa & Stryker 2012). These views have been revised by measures showing that, while cortical responses to the contralateral eye are indeed largely intrinsically established, the emergence of cortical responses to the ipsilateral eye requires patterned vision and their development is competitive (Smith & Trachtenberg 2007, Faguet et al. 2009, Tan et al. 2021). That is, and by contrast to contralateral eye cortical responses, the emergence of ipsilateral eye cortical responses is informed by the pattern of vision through both the ipsilateral eye and the contralateral eye. As discussed below, the relatively delayed development of ipsilateral eye cortical responses places it in a situation where vision and competition dictate its success in establishing a cortical foothold.

### Alternative Model of Vision-Dependent Development of V1

Considering the initial ocular imbalance and the differential impact of vision on the two ocular streams, a different picture emerges of the role of vision in establishing the mature V1. Here, we examine the maturation and maintenance of cortical responses to each eye, identifying differences exploited after eye opening to build optimal neural circuits in V1. We highlight the evolutionary pressures acting specifically on the ipsilateral pathway, emphasizing its greater plasticity potential and central role in optimizing binocular vision during the critical period. Before offering a new model based on these eye-specific differences in development and plasticity, we must first address a key pillar of the current model: inhibition.

### What Is the Role of Inhibition?

The predominant view is that mature inhibition is required for cortical plasticity. Thus, the delayed opening of the critical period mirrors the equally delayed maturation of intracortical inhibition mediated by fast-spiking inhibitory neurons, also referred to as parvalbumin (PV)-expressing neurons or PV cells (Katagiri et al. 2007, Kuhlman et al. 2011, Espinosa & Stryker 2012, Levelt & Hübener 2012, Hooks & Chen 2020, Reh et al. 2020). These PV cells control spiking and gain of local excitatory neurons (Atallah et al. 2012, Polack et al. 2013) and are thought to be necessary for critical period plasticity (Hensch et al. 1998).

The predominant view is that excitation and inhibition are not balanced in the first week after eye opening and this imbalance prevents plasticity. Vision acts to balance this excitation/inhibition (E/I) ratio by strengthening inhibition, and this in turn opens the critical period, enabling vision to now control the plasticity of excitatory neural networks. However, this model is inconsistent with several observations. In vivo recordings show that the E/I ratio at eye opening is no different than at postnatal days 21–28, the classically defined critical period (Fang et al. 2021). Similarly, no E/I imbalance is observed in pyramidal neurons recorded in cortical slices made from precritical period–aged mice (Xue et al. 2014). These measures suggest that as inhibitory cells become more integrated into local circuits—by strengthened excitatory inputs to inhibitory neurons, increased firing rates of inhibitory neurons, and changes in their tuning properties (Kuhlman et al. 2011, 2013; Ma et al. 2013)—the increased inhibition onto local excitatory neurons is continually balanced by equal increases in excitatory strength.

The idea that the maturation of inhibition triggers an opening of the critical period is rooted in studies where inhibition was artificially enhanced, either by administering diazepam, a positive allosteric modulator of the GABA_A_ receptor, or by overexpressing brain-derived neurotrophic factor (BDNF) in pyramidal neurons. In both cases, a precocious critical period was observed (Hanover et al. 1999, Huang et al. 1999, Fagiolini & Hensch 2000, Fagiolini et al. 2004). Complicating this interpretation, more recent studies find that cortical processing is not normal in these animals. For example, the normal development of binocular neurons with nonlinear or complex receptive field tuning does not occur in these mice (Wang et al. 2013, Tan et al. 2021). The formation of such binocular neurons is vision dependent and competitive (Tan et al. 2020), indicating that enhancing inhibition soon after eye opening does not simply switch on the mechanisms that gate synaptic plasticity. An additional concern is that diazepam at the doses used in these studies delays the normal development of cortical response selectivity when stimulated through the ipsilateral eye (Tan et al. 2021). We discuss below why this is problematic.

A revised view of inhibition is that, rather than initiating experience-dependent plasticity, it consolidates excitatory circuit changes driven by experience. Supporting this view, the first change to cortical circuitry after vision loss is the disinhibition of pyramidal neurons in layer (L)2/3, caused by the rapid loss of excitatory input to PV cells (Kuhlman et al. 2013, Ma et al. 2013, Feese et al. 2018). This causes a transient imbalance in the E/I ratio, and the resultant disinhibition opens a window for subsequent excitatory plasticity. Notably, in mutant animals retaining ocular dominance plasticity into adulthood, this same feedforward disinhibition continues to be engaged (Frantz et al. 2020). Although via a different mechanism, transient disinhibition also gates adult cortical plasticity (Froemke et al. 2007, Letzkus et al. 2015).

The rapid and transient disinhibitory effect of vision loss may aid the interpretation of studies showing that the critical period can be reopened in adult V1 via heterochronic transplantation of PV precursor cells into V1 (Southwell et al. 2010, Davis et al. 2015, Hoseini et al. 2019). When transplanted into adult V1, the maturation of these precursor cells follows the same temporal milestones that typify the normal development of native PV cells. The most significant finding from these transplant studies was that a new critical period opens in adult mice when these transplanted cells reach the same cellular age at which endogenous PV cells are thought to trigger the opening of the normal critical period—about 34 days after transplantation. This is taken as evidence that the maturation of PV cells triggers the onset of the critical period. An alternative view, grounded in disinhibition, is that as these new PV cells incorporate into the adult circuit, the synapses that they form on local pyramidal neurons are rapidly and homeostatically balanced by the addition of excitatory synapses. A probable scenario is that the excitatory inputs to these transplanted PV cells, much like those to native PV cells at an earlier age, are susceptible to loss when vision is deprived. The result would be the same transient disinhibition of excitatory networks that promotes plasticity in juveniles.

In our view, the most parsimonious interpretation of the available data is that excitation and inhibition are balanced from eye opening onward and that the strengthening of inhibitory synapses with normal vision consolidates equally strong vision-dependent changes in excitatory synaptic connectivity. Visual deprivation drives a transient loss of feedforward excitation to PV cells, followed by a window during which excitatory inputs are restructured to adequately rebalance the E/I ratio. In the case of monocular deprivation, this rebalancing would involve a reduction of deprived eye inputs and a strengthening of open eye inputs. Thus, transient disinhibition promotes plasticity and, conversely, inhibition promotes consolidation.

### Different Rules Govern the Development and Plasticity of the Contralateral and Ipsilateral Eye Cortical Representation

Cortical responses to vision through the contralateral eye appear to mature intrinsically. At eye opening, measures of ocular responses show that the cortex is almost entirely driven by the contralateral eye (Frégnac & Imbert 1978; Albus & Wolf 1984; Crair et al. 1998, 2001; Tan et al. 2021, 2022). Visually evoked responses first emerge in L4 (Albus & Wolf 1984). And while these responses are not fully mature at eye opening, they already exhibit preferences for orientation, direction, and spatial frequency (Albus & Wolf 1984, Hoy & Niell 2015, Tan et al. 2022).

The requirement for vision in the further maturation of contralateral eye cortical responses differs across species. In mice—where tuning in the thalamorecipient L4 and supragranular L2/3 is roughly equivalent—the numbers of responsive neurons, as well as measures of orientation selectivity, spatial frequency, and response linearity, continue to mature even when animals are raised in the dark, indicating that intrinsic mechanisms account for much of this maturation (Kuhlman et al. 2011; Li et al. 2012; Tan et al. 2020, 2021). In kittens, as well, cortical responses to the contralateral eye mature in the absence of vision (Sherk & Stryker 1976), though some reports indicate that while the initial maturation of tuning is hardwired, fully adult-like response tuning requires vision (Frégnac & Imbert 1978).

In ferrets, vision is required for normal development of contralateral eye responses in supragranular layers, but not L4. When ferrets are reared in complete darkness, orientation tuning matures but does not reach adult levels. However, if both eyelids are sutured, permitting some poor-quality vision, orientation tuning fails to mature (White et al. 2001). A comprehensive laminar electrophysiological examination of receptive field tuning from lid-sutured ferrets showed that the quality of early visual experience determines tuning in L2/3, while tuning in L4 is intrinsically established (Chapman & Stryker 1993).

A reasonable conclusion from these studies is that the tuning of contralateral eye cortical responses is mostly intrinsically established, with vision needed to maintain this tuning—potentially more so in L2/3. The intrinsic nature of L4 tuning may have its origins in the retinal epithelium. Computational modeling studies suggest that many receptive field tuning properties in L4 can emerge from a random sampling of local ON and OFF channels originating in the mosaic of ON-center and OFF-center ganglion cells in the retina (Ringach 2004, Paik & Ringach 2011). Supporting a permissive role of vision in this process, experimental suppression of ON-center retinal ganglion cell activity in early postnatal ferrets prevents the emergence of these receptive field tuning properties (Chapman & Gödecke 2000). In vivo whole-cell recordings of L4 neurons under conditions where intracortical activity is suppressed provide direct evidence that this tuning is retinothalamic in origin—L4 neurons are as well-tuned when driven solely by thalamocortical inputs as when intracortical activity is intact (Ferster et al. 1996, Lien & Scanziani 2018).

Once cortical responses to the contralateral eye gain sufficient strength—about a week after eye opening in mice and kittens—the maintenance effect of vision becomes clear. In kittens raised with binocular lid suture, cortical responses to the contralateral eye strengthen normally for about the first week after the age of natural eye opening. However, if visual deprivation continues, these responses regress back to the weaker representation typically seen at eye opening (Crair et al. 1998).

While vision is needed to maintain the contralateral eye cortical response, this maintenance may not be competitive. That is, the presence or absence of vision through the ipsilateral eye does not appear to significantly impact the development or plasticity of the contralateral eye representation. In mice, where this has been directly examined, the loss of cortical responsiveness to the contralateral eye is the same whether only the contralateral eye or both eyes are sutured shut at the peak of the critical period. This contralateral solipsism is further evident when only the ipsilateral eye is shut: The cortical response to the contralateral eye does not get stronger—it remains the same (Faguet et al. 2009). Thus, the maintenance of the cortical representation of the contralateral eye is governed solely by the quality of vision through that eye; it is not competitive.

By contrast, cortical responses to the ipsilateral eye mature later than those to the contralateral eye, and their development is sensitive not only to the quality of vision through the ipsilateral eye but through the contralateral eye as well (Smith & Trachtenberg 2007).

When only the contralateral eye is sutured to prevent natural eye opening, leaving spontaneous activity intact, the number of neurons responsive to the ipsilateral eye fails to reach normal adult levels even though vision is perfectly normal through the ipsilateral eye. Moreover, the receptive field tuning profiles of the few neurons that do respond to the ipsilateral eye lack normal selectivity. This indicates that a mismatch in activity between the eyes impairs the emergence of cortical responsiveness to the ipsilateral eye (Tan et al. 2021). The influence of the contralateral eye on ipsilateral eye cortical maturation is further made clear by experiments showing that the cortical representation of the ipsilateral eye matures faster if retinal activity in the contralateral eye is silenced (Smith & Trachtenberg 2007). Thus, in stark contrast to what we saw for the contralateral eye's cortical representation, the development of cortical responses to the ipsilateral eye is both vision dependent and competitive—its maturation is regulated by the quality and magnitude of both its own activity and that of the contralateral eye.

This difference in the rules governing plasticity serves an important behavioral purpose, which we discuss below, and explains the illusion of a precritical period. To reiterate, the prevailing view is that, in mice and cats, there is a period spanning about a week after eye opening when monocular deprivation does not alter ocular dominance scores. This finding was taken as evidence for an onset to the critical period. We now understand that this conclusion is erroneous. When the contralateral eye is closed within this first week after eye opening, cortical responses to the contralateral eye mature relatively normally. However, ipsilateral eye responses are impaired and do not strengthen as they should, even though the ipsilateral eye has normal vision (Smith & Trachtenberg 2007, Tan et al. 2021). Because of this paradoxical effect, closing the contralateral eye does not result in a shift in ocular dominance toward the open, ipsilateral eye. Instead, a contralateral bias resembling that of the normal immature cortex persists. Even though vision is greatly impacting circuit development from the moment the eyes open, this impact is missed by measures of ocular dominance plasticity.

### The Establishment of Binocular Neurons

How does the temporal mismatch in the development of contralateral and ipsilateral eye responses, along with the uniquely competitive and vision-dependent plasticity of ipsilateral eye responses, inform normal development? As noted above, the most physiologically salient consequence of the critical period is the establishment of binocular neurons with matched receptive field tuning properties (Ohzawa et al. 1996, Wang et al. 2010, Cang et al. 2023). These neurons are sensitive to absolute disparity, and their disparity tuning is used to extract information about depth (Anzai et al. 1999a,b; Cumming & DeAngelis 2001; Parker 2007). The conventional view had been that improvements in binocular matching and tuning selectivity occur by sculpting the tuning of these neurons from broad and mismatched to sharp and matched (La Chioma & Hübener 2021).

Longitudinal in vivo recordings offer an alternative model where binocular neurons are continuously formed via the conversion of monocular neurons (Tan et al. 2020, 2021), while some initially binocular neurons lose input from one eye and become monocular. Thus, the binocular pool is in continuous flux from eye opening until the closure of the critical period. This flux is driven by the development and refinement of ipsilateral eye cortical responses.

At eye opening, most neurons are responsive to the contralateral eye. Responses to the ipsilateral eye emerge thereafter; their tuning is initially much poorer than that of contralateral eye evoked responses but improves considerably over the next few weeks if vision is normal. Many neurons that were initially responsive solely to the contralateral eye at eye opening then gain inputs from the ipsilateral eye and become binocular. Because ipsilateral eye tuning is relatively poor in the first few days after eye opening, the first binocular neurons to form reflect this poor tuning. As vision improves the tuning of ipsilateral eye responses, better-tuned binocular neurons are established while many of the earlier-formed but poorly tuned binocular neurons lose input from one eye and exit the binocular pool (Tan et al. 2020). The result of this iterative process is a pool of highly selective binocular neurons where the selectivity of responses to each eye is precisely matched. This process occurs first in simple cells and later in complex cells (Wang et al. 2013, Gu & Cang 2016, Tan et al. 2020). Notably, these instructive effects of vision appear to be most pronounced in L2/3. Other mechanisms may operate in parallel to build binocular circuitry (Wang et al. 2010, Gu & Cang 2016, Chang et al. 2020, La Chioma & Hübener 2021).

There is a notable similarity in the processes of binocular matching and ocular dominance plasticity. In both cases, the weaker input loses. In binocular matching, when the receptive field tuning properties of the two eyes do not match, the weaker eye is eliminated (Tan et al. 2020). In ocular dominance plasticity, where the receptive field tuning is artificially mismatched by unilateral lid suture, the weaker, deprived eye also loses. The most parsimonious observation is that ocular dominance plasticity is simply an epiphenomenon of binocular matching.

A consequence of this selection process where binocular neurons are continually established and tested by vision is that the final pool of binocular neurons is significantly more selective for orientation and tuned to higher spatial frequency stimuli than the monocular pool of neurons. This selectivity aids in foraging and defense because these highly selective neurons are mapping the visual field directly in front of the animal.

### Evolution Preferentially Impacts Ipsilateral Eye Projections

Throughout this critical look at the critical period, we have argued that experience acts differentially and preferentially upon the development of cortical representation of the ipsilateral eye. Given that evolution operates through development (Gould 1985), we should expect the ipsilateral eye pathway to be significantly affected by evolutionary pressures.

Plastic systems exhibit greater variance in gene expression (Gray & Spiegel 2019, Spiegel 2021, Griffith et al. 2024), and such variance is the lever used by evolutionary pressures to alter developmental trajectories in the nervous system (Hardingham et al. 2018). Therefore, plastic systems, and in this case the ipsilateral eye pathway, should experience the most alterations during evolution. Indeed, there is ample evidence for this (Larsson 2011, 2013).

Evolutionary pressures have shaped retinal output by preferentially acting on retinal ganglion cells (Hahn et al. 2023) and modifying their ipsilateral projections through guidance cues in the optic chiasm (Herrera et al. 2024). An intriguing hypothesis is that stereopsis evolved to improve eye-forelimb coordination. This is particularly evident in primates, which evolved a manual foraging system, but it is also apparent in any animal that can see its forelimbs and uses them for visually guided reaching or grasping tasks. A natural consequence of this hypothesis is that stereopsis in any given species is optimized for distances within arm's length (Richards 2009, Volcic et al. 2013).

The cortical regions responsible for motor control and sensory perception of limb movements are situated in the cerebral hemisphere opposite to the limb being used. Thus, a second consequence of the hypothesis is that in animals that use their forelimbs frontally—where the limb is seen by both eyes simultaneously—it is necessary to have an ipsilateral projection for the retinal ganglion cells mapping central visual space in order to preserve hemispheric correspondence of visuomotor feedback (Larsson 2011, 2013). That is, if you reach out with your right hand to grasp an object in front of you, you are controlling your right hand with your left motor cortex, yet both eyes will view your right hand and the object you are trying to grasp. To accurately grasp the object, the left hemisphere, which is controlling the forelimb in this case, must integrate binocular visual information with proprioceptive information to accurately guide motor commands. This requires an ipsilateral projection from the left eye in addition to the typical contralateral projection from the right eye to convey binocular disparity information about the central visual field to the left hemisphere where the motor movements are planned and executed.

Cross-species differences in the proportion of ipsilaterally projecting retinal ganglion cells and their retinal positioning reflect these evolutionary pressures. For example, in mice, the few existing ipsilaterally projecting retinal ganglion cells map the upper portion of the central visual field (Dräger & Olsen 1980, Johnson et al. 2021). Their positioning reflects the visual obstruction caused by the snout, which restricts viewing of the contralateral paw to this region of visual space. Indeed, when mice grasp for prey during the final stage of hunting, they lower their heads to position the prey and the forelimb in this visual region (Holmgren et al. 2021). Despite their small number of ipsilaterally projecting retinal ganglion cells, mice have binocular neurons in V1 tuned to disparity, providing information well-suited for behavioral tasks that rely on depth discrimination (Boone et al. 2021). In summary, there is a clear correlation between the ipsilateral projection's susceptibility to evolutionary pressures and its enhanced experiential plasticity during development.

### Molecular Mechanisms Underlying the Critical Period

In this review we have noted that a mechanistic understanding of ocular dominance plasticity faces challenges similar to those that once burdened the field of long-term potentiation in that almost every conceivable mechanism has been implicated (Sanes & Lichtman 1999). However, certain pathways and molecules stand out in their significance. The critical period involves two main components: (*a*) the experience-dependent construction and maintenance of higher-order feature detectors, including complex binocular neurons, and (*b*) the closure of this period of plasticity. Essentially, these are two sides of the same coin. Two models merit discussion.

#### Model 1: Synaptic plasticity at thalamorecipient synapses.

One model of critical periods focuses on changes to synaptic plasticity at thalamorecipient synapses. In primary visual and somatosensory cortices, experience leads to the progressive loss of *N*-methyl-d-aspartate (NMDA) receptors, and thus the loss of plasticity at thalamorecipient synapses (Carmignoto & Vicini 1992, Crair & Malenka 1995, Kirkwood et al. 1995, Yaeger et al. 2024). Thus, synaptic plasticity is initially high, and visual experience suppresses this plasticity as synaptic connectivity is refined. This loss of plasticity closes the critical period. Restricting this consolidation to thalamorecipient synapses may solve the plasticity-stability dilemma by enabling cortical networks to indelibly store information—such as basic receptive field tuning properties—at some synaptic sites while maintaining plasticity at other sites to support life-long learning (Yaeger et al. 2024).

It remains unclear whether experience first drives changes in these synapses or if plasticity originates within cortex and subsequently instructs changes to thalamic input. For instance, feedback from L6 to the thalamus could convey cortically initiated changes back to the thalamus, altering its activity patterns (Weliky & Katz 1999). This could restructure thalamocortical connectivity to reflect this shift in activity. In this case, plasticity at thalamocortical synapses is downstream of and amplifies cortically initiated synaptic plasticity. Loss of plasticity at thalamocortical synapses would then create a permanent memory of experience-dependent plasticity. Supporting the view that thalamocortical plasticity is downstream of cortical plasticity, neither plasticity at thalamocortical synapses nor changes in protein synthesis in thalamic relay cells are required for the expression of cortical plasticity (Taha & Stryker 2002, Frantz et al. 2020). However, for cortical plasticity to become permanent, as occurs during the critical period, it must alter the connectivity of thalamic input.

#### Model 2: Cortical disinhibition as a driver of plasticity.

A second model posits that cortical disinhibition initiates experiential plasticity. Various studies agree that feedforward excitatory inputs from L4 to PV interneurons in L2/3 gate cortical plasticity. As discussed above, the loss of cortical responsiveness to an eye deprived of vision is preceded by, and depends upon, the disinhibition of L2/3 pyramidal neurons (Kuhlman et al. 2013, Frantz et al. 2020).

Notably, plasticity at the synapse from L4 excitatory neurons to L2/3 PV cells appears to be regulated by the expression of the Nogo-66 receptor (NgR1) in L4 excitatory neurons. NgR1 is a protein containing leucine-rich repeats and is tethered to the extracellular surface by a lipid anchor (Stephany et al. 2016, Frantz et al. 2020). Eliminating this receptor from L4 is sufficient to sustain adult cortical plasticity at levels seen in juveniles.

Thus, the primary impact of visual deprivation during the critical period appears to be on the firing rates of L2/3 pyramidal neurons, which are disinhibited as excitatory synapses from L4 to L2/3 PV cells are lost. This may explain why vision seems to preferentially inform the establishment of connectivity and higher-level feature detectors in L2/3 (Ko et al. 2013, Tan et al. 2020) and even the fundamental transcriptomic identity of glutamatergic neurons in L2/3 (Cheng et al. 2022). In this sense, vision quite literally shapes the identity of L2/3 pyramidal neurons. Compared to other glutamatergic populations in the cortex, L2/3 neurons have proven most susceptible to evolutionary pressures and are more numerous in primates (Berg et al. 2021, Galakhova et al. 2022).

## CONCLUDING REMARKS

Evolution has established preferred sites—or pathways—of plasticity at various levels of biology. In the human genome, segmental duplications act as hotspots for genetic variation, leading to the emergence of novel genes and altered copy numbers. In the neocortex, evolution and development preferentially mold the functional properties and transcriptomic identities of glutamatergic neurons in layers 2/3. Crucially, the cortical representation of the ipsilateral eye appears to be preferentially instructed by vision and binocular competition. This plasticity serves to establish complex binocular neurons and disparity tuning in V1—features fundamental to the emergence of depth perception and the proper execution of visually guided reaching and grasping tasks.

Over a decade and a half ago, Jianhua Cang and colleagues (Wang et al. 2010) redefined the critical period. Rather than framing it as a period during which the cortex is susceptible to destructive processes, they showed that vision is acting constructively. Vision builds higher-order feature detectors that cannot be established intrinsically (Kowalewski et al. 2021). Longitudinal studies then revealed that vision continually assembles and disassembles functional networks of new feature detectors as inputs are refined and more accurate information is conveyed to the cortex (Tan et al. 2020). In doing so, functional networks are not simply refined but fundamentally reconfigured by our experiences.

Building upon this view, we do not subscribe to the notion that there are successive waves of critical periods that open and close at different times across different cortical areas. Plasticity is not passed from one region to the next like a baton. Instead, the entire cortex begins learning about the outside world from the moment thalamic inputs first relay sensory and proprioceptive information from the periphery. These cortical networks are subsequently reconfigured to optimize sensation and behavior, given the constraints of the sensory organs and musculoskeletal system. We deviate from conventional views by asserting that this learning is restricted to specific circuit elements. As this learning continues, the cortex uses these memories to make predictions about future events and directs its metabolic resources toward dealing with unexpected occurrences (Keller & Mrsic-Flogel 2018, Jordan & Keller 2023). The consolidation of thalamocortical input at the closure of the critical period makes permanent the fundamental receptive field tuning properties used to build more sophisticated feature detectors and conjunctions necessary for successful behaviors, while the continuation of intracortical plasticity after critical period closure ensures lifelong learning (Yaeger et al. 2024). In essence, the critical period reflects the brain's sprint to integrate sensory experiences into its developing architecture while ensuring that the fundamental processing necessary for perception—tuned to the demands of the environment—is permanently inscribed in primary sensory cortices. This, in turn, provides the stable foundation needed for perception and action to be continually adapted to environmental demands throughout life.

## SUMMARY POINTS

1. The critical period begins at eye opening. There is no window of plasticity, but rather a continuous state of plasticity that begins with spontaneous retinal activity and continues after eye opening until thalamocortical plasticity ends, consolidating feedforward connectivity.
2. Inhibition similarly suppresses changes to the excitatory circuitry in the cortex, and reductions in inhibition are needed for plasticity to resume.
3. The function of neocortical processing is improved by population drift—the swapping of cells into and out of networks as a function of their tuning properties rather than a change in the tuning of the initial population.
4. The ipsilateral and contralateral eye cortical representations develop at different rates and follow different rules of experience-dependent plasticity, with the ipsilateral eye representation being the most plastic.
5. This plasticity of the ipsilateral eye pathway reflects evolutionary pressures on this pathway.
6. Vision-dependent changes in cortical function during the critical period appear to emerge in layer (L)2/3, likely at L4 to L2/3 synapses.

## FUTURE ISSUES

1. Future research should take a more comparative approach and ask which cell types have been most impacted by evolutionary pressures. Doing so will likely reveal preferential sites of experience-dependent plasticity. Once those are known, then the signaling pathways can be worked out in those cell types.
